# Phase Diagrams and Thermal Properties of Fatty Acid Ternary Eutectic Mixtures for Latent Heat Thermal Energy

**DOI:** 10.3390/ma19020356

**Published:** 2026-01-16

**Authors:** Dongyi Zhou, Fanchen Zhou, Jiawei Yuan, Zhifu Liu, Yicai Liu

**Affiliations:** 1 School of Energy and Mechanical Engineering, Hunan University of Humanities, Science and Technology, Loudi 417000, China; jufang802884@163.com (F.Z.); y951723@163.com (J.Y.); 2 Ningbo Longhui Electromechanical Engineering Co., Ltd., Ningbo 315000, China; lhjd998@163.com; 3 School of Energy Science and Engineering, Central South University, Changsha 410083, China

**Keywords:** fatty acid, ternary eutectic mixtures, phase diagrams, thermal properties

## Abstract

This study utilized capric acid (CA), lauric acid (LA), myristic acid (MA), palmitic acid (PA), and stearic acid (SA) as alternative feedstocks to conduct theoretical analyses on ten fatty acid-based ternary eutectic systems. By leveraging the Schrader equation, phase diagrams for each system were constructed, and their theoretical eutectic points were calculated. The CA-LA-MA (capric acid–lauric acid–myristic acid) ternary system was selected as a representative for experimental fabrication: differential scanning calorimetry (DSC) was employed to characterize its thermal properties, while Fourier transform infrared spectroscopy (FT-IR) and thermogravimetric analysis (TGA) were used to assess its functional group composition and thermal stability, respectively. Theoretical calculations indicate that the ten ternary eutectic systems exhibit melting temperatures ranging from 17.11 °C to 37.61 °C, with phase change latent heats spanning 167.8 J·g^−1^ to 189.6 J·g^−1^. For the CA-LA-MA system, experimental DSC results confirm that its eutectic melting temperature is 16.0 °C (accompanied by a phase change latent heat of 177.0 J·g^−1^, with minor deviations from theoretical predictions attributed to reagent impurities and operational errors). TGA characterization further reveals that the CA-LA-MA mixture has an initial weight loss temperature (corresponding to ~1% mass loss) of 115.6 °C and an extrapolated onset weight loss temperature of 164.8 °C, confirming reliable thermal stability below 100 °C—consistent with its low-temperature application design. These results validate the consistency between theoretical predictions and experimental data, and demonstrate that fatty acid-based ternary eutectic mixtures are promising candidates for low-temperature thermal energy storage applications.

## 1. Introduction

Amid the global energy transition to decarbonization, efficient energy storage and cascade utilization are core bottlenecks for resolving supply–demand spatiotemporal mismatches and boosting renewable energy absorption [1]. IEA statistics show renewable energy accounted for 28% of global power generation in 2023 [2]. However, volatility of wind and solar power has limited their actual absorption rate to below 60% [3], with massive energy waste caused by the lack of efficient thermal energy storage technologies. Phase change thermal energy storage (PCTES) technology, which utilizes latent heat during solid–liquid phase transition, enables efficient energy utilization and is widely applicable in low-temperature scenarios such as building energy conservation and industrial waste heat recovery [4,5,6,7,8]. Solid–liquid phase change materials (PCMs) are the core of PCTES, with organic PCMs (e.g., fatty acids) attracting significant attention due to their good chemical stability, low corrosiveness, and controllable phase change process [9,10], superior to inorganic PCMs that suffer from corrosiveness and phase separation [11].

As natural organic PCMs, fatty acids (FAs) feature wide sources, biodegradability, and moderate phase change enthalpy (150–250 J·g^−1^) [12], but single fatty acids are limited by fixed phase change temperatures, supercooling, and poor long-term cycling stability [13]. For instance, stearic acid (phase change temperature 69.4 °C) is incompatible with 50 °C waste heat recovery [14], while unsaturated fatty acids (e.g., oleic acid, 13 °C) are prone to oxidation [15]. In addition, the phase change temperature of a single component is difficult to meet the requirements of diverse application scenarios (e.g., building heating at about 20–30 °C and electronic cooling at 40–60 °C) [16,17]. Multicomponent compounding of fatty acids to form eutectic systems has thus become an effective strategy to tune phase change temperatures, reduce supercooling, and improve stability [18,19].

Binary fatty acid eutectic systems have been widely studied. Yuan et al. [20] prepared a palmitic acid (PA)–stearic acid (SA) eutectic (58.8:41.2, mass ratio) with a phase change temperature of 53.95 °C. Sari et al. [21] fabricated a myristic acid–stearic acid eutectic (64:36, mass ratio) with a melting point of 44.13 °C. Zhou et al. [7] developed binary eutectics with phase change temperatures of 17.7–57.1 °C, suitable for low-temperature storage. Despite these advances, binary systems have inherent drawbacks: their phase change temperature adjustable range is constrained by the intrinsic properties of the two components, and they often exhibit latent heat attenuation during long-term cycling [22]. Fan et al. [23] reported that a lauric acid (LA)–MA binary system (70:30 mass ratio), despite an initial phase change latent heat of 166.18 J·g^−1^, underwent gradual latent heat degradation with repeated thermal cycles, which impairs its long-term application in building energy conservation.

Ternary fatty acid mixtures overcome these limitations by introducing a third component, which broadens the phase change temperature adjustable range (10–80 °C) and enhances stability through synergistic intermolecular interactions between the three components [24]. Several studies have validated the feasibility of ternary fatty acid eutectics. Du et al. [25] prepared a capric acid (CA)–SA–PA ternary eutectic (77.4:8.6:14.0) with a phase change temperature of 18.60 °C and stable performance after 500 cycles. Dai et al. [26] developed 13 fatty acid-based ternary eutectics (25.62–63.84 °C, 152.44–231.52 J·g^−1^) with excellent stability via Schrader equation-based eutectic point prediction. Despite these advances, ternary systems still face critical challenges: inadequate phase diagram databases for rational design [27], insufficient long-term stability due to component volatilization/oxidation [28], and low thermal conductivity [29]. Notably, research on the stability of fatty acid eutectics remains fragmented, with ambiguous evaluation methodologies and unspecified characterization time scales—two critical knowledge gaps that impede their practical deployment [30,31]. For instance, although Du et al. [25] documented the stability of fatty acid eutectics over 500 thermal cycles, systematic investigations into long-term storage stability (e.g., on monthly or yearly time scales) and standardized evaluation protocols (e.g., thermogravimetric analysis (TGA) for thermal decomposition stability, Fourier-transform infrared spectroscopy (FT-IR) for chemical structural stability, and cyclic differential scanning calorimetry (DSC) for phase-change performance retention) are still scarce.

Phase diagrams are critical for guiding ternary PCM composition design, as they clarify eutectic point distribution and support performance prediction [32]. However, systematic construction of ternary fatty acid phase diagrams and analysis of the structure-activity relationship between phase change properties and composition are extremely scarce [33]. Most existing studies focus on binary systems or empirical optimization of specific ternary ratios, lacking generalized rules, which leads to over-reliance on trial-and-error experiments and hinders the industrialization of ternary fatty acid PCMs.

To address these gaps, this study selected five typical fatty acids (capric acid, lauric acid, myristic acid, palmitic acid, and stearic acid) as raw materials, considering their favorable properties: phase change temperatures of 20–70 °C (tunable to 17.11–37.61 °C via eutectic regulation), high latent heat, good stability, and low cost, which make them ideal for eco-friendly PCMs. This work clarifies the composition-dependent regulations of phase change properties and systematically evaluates the thermal performance and thermal stability of the prepared eutectic mixtures. The findings are expected to enrich the fundamental database of fatty acid-based PCMs and provide theoretical and technical support for the development of low-cost, high-performance materials for latent heat thermal energy storage.

## 2. Materials and Methods

### 2.1. Materials

All fatty acid reagents including capric acid (CA), lauric acid (LA), myristic acid (MA), palmitic acid (PA) and stearic acid (SA) with a purity grade of ≥98.5% were sourced from Shanghai Zhanyun Chemical Co., Ltd. (Shanghai, China). The thermal performance parameters of the fatty acid reagents, including melting temperature (*T*_m_), melting peak temperature (*T*_mp_), melting enthalpy (Δ*H*_m_), freezing temperature (*T*_f_), freezing peak temperature (*T*_fp_), freezing enthalpy (Δ*H*_f_), solid-state specific heat capacity (*C*_p,s_) and liquid-state specific heat capacity (*C*_p,l_), are summarized in Table 1.

### 2.2. Theoretical Prediction of Eutectic Properties

To accurately predict the eutectic mass ratios and onset melting temperatures of binary and multi-component fatty acid eutectics, and provide a theoretical basis for experimental formulation design, the core process of theoretical prediction is elaborated as follows: The ideal eutectic phase equilibrium model was adopted for theoretical prediction, considering that fatty acid molecules interact mainly via weak van der Waals forces and their mixing behavior conforms to the ideal solution assumption in the eutectic-forming concentration range. This model reliably fits the phase equilibrium laws of fatty acid eutectics for composition and melting temperature prediction.

The core input parameters are the melting temperature (*T*_m_) and enthalpy of fusion (Δ*H*_m_) of pure fatty acid components, which directly affect prediction reliability. *T*_m_ and Δ*H*_m_ of pure components (CA, LA, MA, PA and SA) were obtained via DSC tests (see Experimental Characterization for details), ensuring consistency with the reagents used in this study.

The prediction was implemented step by step from binary to multi-component systems: (1) Binary system prediction: Substitute verified *T*_m_ and Δ*H*_m_ into the ideal eutectic phase equilibrium equation to solve for the theoretical eutectic mass ratio and onset melting temperature; (2) Multi-component system prediction: Extend the binary model to ternary (e.g., CA-LA-MA) and above systems via the “successive approximation method”—fix the eutectic ratio of two components, solve for the optimal ratio of the third, and optimize the overall ratio iteratively to obtain the theoretical results; (3) Result correlation: Use the theoretically predicted parameters as the basis for eutectic sample preparation and thermal performance testing, realizing the connection between theoretical prediction and experimental verification.

Section 3.1 will elaborate on the specific calculation process using the Schrader Equation to clarify the quantitative implementation of theoretical prediction.

### 2.3. Preparation of Ternary Fatty Acid Mixtures

This study aimed to prepare ternary eutectic mixtures using three selected components from five alternative monounsaturated fatty acids, namely CA, LA, MA, PA, and SA. First, the weighed acids were dried in a DZF-6020B vacuum drying oven (Hangzhou Lipei Instrument Tech. Co., Ltd., Hangzhou, China, 220 V/50 Hz, 1100 W) at 60 °C (0.09 MPa) for 3 h to remove adsorbed moisture. The dried materials were mixed in a preset proportion in a 100 mL beaker, then heated to full melting (≈15 min) in a HCJ-2 magnetic stirring water bath (Hangzhou Lipei Instrument Tech. Co., Ltd.) (60 °C), with stirring at 300 r·min^−1^ for 30 min (PTFE stir bar, 30 mm) to homogenize. After stirring, the beaker mouth was tightly sealed with a piece of clean plastic wrap, then transferred to a 4 °C laboratory refrigerator (temperature fluctuation: ±2 °C) and held for 30 min to achieve complete solidification (no phase separation observed visually). Finally, the solidified mixture was vacuum-sealed in plastic bags and stored at 4 °C for subsequent characterization.

### 2.4. Characterization

The thermal properties of the samples were characterized using a differential scanning calorimeter (DSC, NETSZCH 214 Polyma, NETZSCH-Gerätebau GmbH, Selb, Germany). Prior to DSC testing, we calibrated the instrument’s temperature axis and heat flow axis using indium standard. The calibration process strictly followed the vendor-recommended procedures to ensure deviations in phase transition temperature and latent heat are ±0.1% and ±4%, respectively. The thermal performance measurements were conducted under a 0.5 bar nitrogen atmosphere. Prior to testing, high-purity nitrogen (≥99.99%) was used to purge the sample chamber at 50 mL·min^−1^ for 30 min to remove residual air, and the pressure was stabilized at 0.5 bar via a calibrated pressure-regulating valve and the instrument’s automatic control module The temperature range was set from −10 °C to 60 °C, with a heating and cooling rate of 10 °C·min^−1^. All DSC tests included blank baseline experiments (empty crucible run under the same heating/cooling program), and the sample curves were corrected by subtracting the baseline using software—specifically, a linear baseline fitted based on the stable regions before the onset and after the end of the phase transition peak—to eliminate instrument drift and background heat capacity effects. All phase change temperatures in this paper (including melting temperature *T*_m_ and freezing temperature *T*_f_) are defined and characterized using the onset temperature of DSC curves, so as to ensure the objectivity and comparability of the data.

FT-IR spectra were recorded using a Thermo Scientific Nicolet iS5 spectrometer (Thermo Fisher Scientific, Waltham, MA, USA) equipped with an attenuated total reflectance (ATR) accessory. Spectral acquisition was performed over a range of 4000–400 cm^−1^ at a resolution of 4 cm^−1^, with 32 scans per spectrum. A background scan was conducted prior to sample measurement to eliminate interference from ambient air and instrument noise. The resulting spectra were processed using OMNIC software (Version 7.3) to obtain absorbance values.

The thermal resistance and thermal stability of the samples were evaluated using Thermogravimetric Analysis (TGA, TA TGA5000IR, TA Instruments, New Castle, DE, USA). The instrument temperature was calibrated using standard reference materials, including indium oxide (In_2_O_3_) and tin (Sn)/zinc (Zn), under a heating rate of 10 °C/min in a nitrogen atmosphere, ensuring the deviation between the measured temperature and literature values was less than ±2 °C. The balance sensitivity was calibrated using standard weights, with mass measurement errors controlled within ±0.05%. All tests were preceded by a blank baseline experiment (empty Al_2_O_3_ crucible) conducted under identical conditions. The sample curves were corrected by subtracting the blank baseline via the instrument software to eliminate background interference.

## 3. Results and Discussion

### 3.1. Phase Diagram of the Ternary Eutectic System

Fundamental thermodynamic and phase equilibrium theories provide a rigorous framework for predicting eutectic mixture composition and thermal performance. In solid–liquid equilibrium systems without liquid infiltration into the solid phase, component fugacities in both phases are equal. By neglecting solid–liquid volume and heat capacity disparities, and substituting triple-point with melting-point temperature, the Schrödinger equation is derived from the system’s phase equilibrium thermodynamics, as shown below [18]:

(1) $$x_{i}=\exp\left[\frac{\Delta H_{mi}}{R}\left(\frac{1}{T_{mi}}-\frac{1}{T_{m}}\right)\right],$$

Herein: *x*_i_ is the molar percentage composition of component *i* (%) ($\sum x_{i}=1$), Δ*H*_mi_ stands for the latent heat of fusion of *i*-th component (in J·mol^−1^), *R* is the gas constant with a fixed value of 8.315 J·(mol·K)^−1^, *T*_mi_ indicates the melting point of component *i* (K), while *T*_m_ denotes the melting point temperature of the mixture (K).

For a binary fatty acid eutectic system with components A and B, Formula (1) can be written as the following Formula (2) [34].

(2) $$\left\{\begin{array}{l} -\frac{\Delta H_{mA}}{T_{mA}}(T_{m}-T_{mA})-RT_{m}\ln(1-x_{A})=0\\ -\frac{\Delta H_{mB}}{T_{mB}}(T_{m}-T_{mB})-RT_{m}\ln(1-x_{B})=0 \end{array}\right.,$$

where Δ*H*_mA_ and Δ*H*_mB_ represent molar latent heat of fusion for the component A and component B, respectively (in J·mol^−1^); *T*_mA_, *T*_mB_ and *T*_m_ correspond to the melting temperature of the component A and component B and their blended mixtures, respectively (in K); *x*_A_ and *x*_B_ denote the mole fraction of the component A and component B, *x*_A_ + *x*_B_ = 1.

Equation (3), which is applied for calculating the melting temperature of the eutectic mixture, can be derived via the simplification of Equation (2).

(3) $$\left\{\begin{array}{l} T_{m}=1/\left(\frac{1}{T_{mA}}-\frac{R\ln x_{A}}{\Delta H_{mA}}\right)\\ T_{m}=1/\left(\frac{1}{T_{mB}}-\frac{R\ln x_{B}}{\Delta H_{mB}}\right) \end{array}\right.,$$

The latent heat of fusion (Δ*H*_m_, J/mol) of fatty acid eutectic systems can be derived based on classical phase equilibrium principles and eutectic formation theories, which is expressed by the following Equation (4) [34]:

(4) $$\Delta H_{m}=T_{m}\sum_{i=1}^{n}\left(\frac{x_{i}\Delta H_{mi}}{T_{mi}}\right),$$

**Figure 1:**
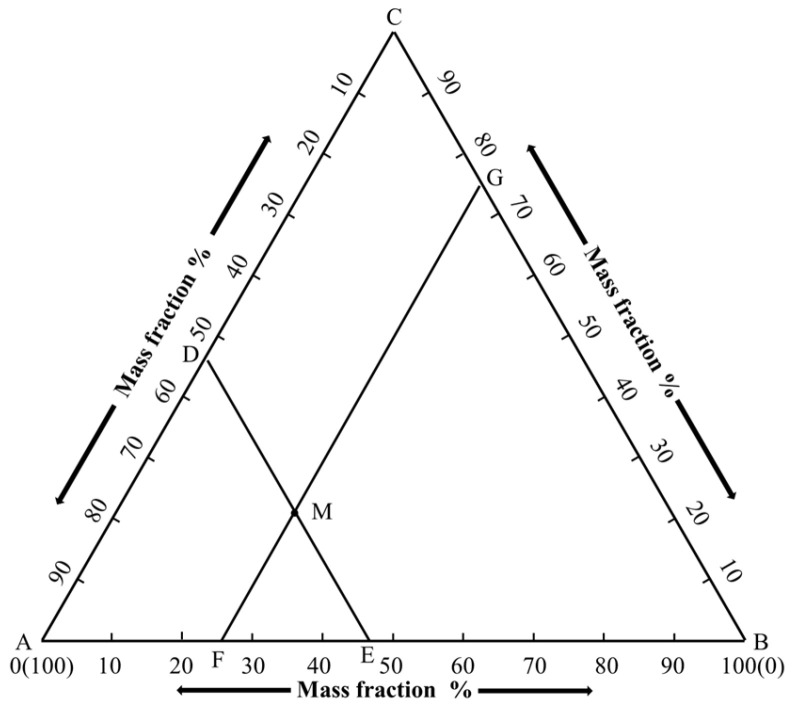
Composition representation of equilateral triangle.

The composition of a ternary system is generally represented by an equilateral triangle, referred to as a composition triangle (also known as a concentration triangle or Gibbs triangle), as shown in the equilateral triangle ABC in Figure 1. In the figure, the three vertices A, B, and C of ΔABC represent pure components A, B, and C (each with a content of 100%), respectively. Points on each side of the triangle denote the relative contents of the two components in the corresponding binary system. Any point inside the triangle represents the composition of a specific ternary system. For example, take point M inside the equilateral triangle: draw DE||BC and FG||AC through point M. Let a, b, and c represent the mass percentages of components A, B, and C, respectively. Then a = BE, b = AF, c = FE, and a% + b% + c% = 1.

**Figure 2:**
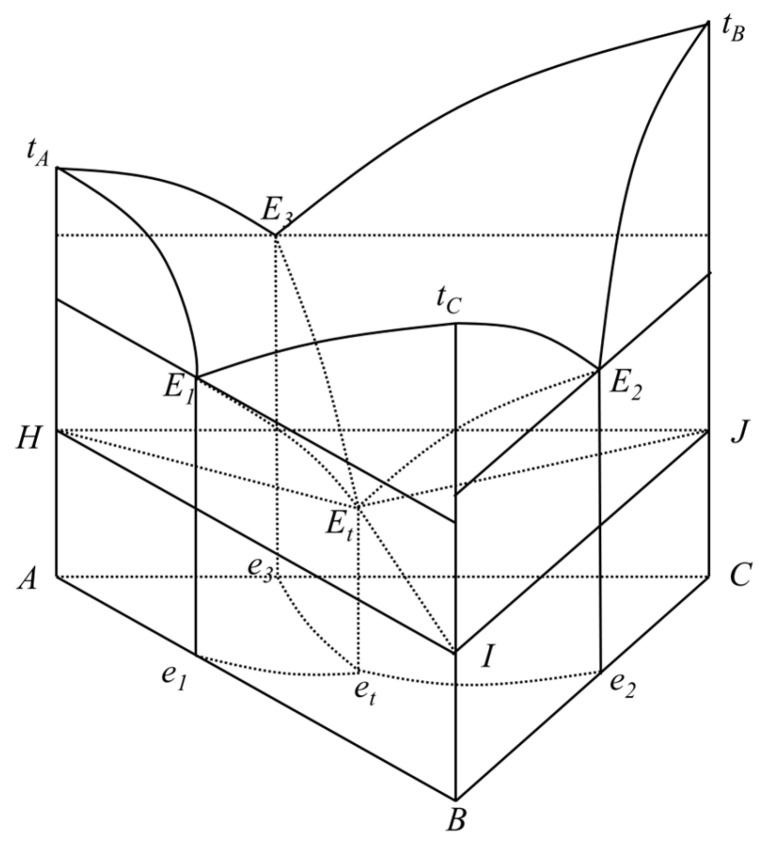
Phase diagram of ternary system with lowest co-melting point.

The ternary solid–liquid phase diagram takes the concentration triangle as the base, with the height perpendicular to the base serving as the temperature coordinate. The entire diagram forms a triangular prism, as illustrated in Figure 2. The three lateral faces represent three binary systems (A-B, B-C, and C-A) each with a binary eutectic point, denoted as *E*_1_, *E*_2_, and *E*_3_, respectively. *t*_A_, *t*_B_, and *t*_C_ are the melting points of the pure components A, B, and C, while Et is the minimum ternary eutectic point. As illustrated in Figure 2, this 3D ternary phase diagram clearly demarcates the single-phase liquid region (above the curved surface of *E*_1_, *E*_2_, and *E*_3_ and *E*) from the solid–liquid coexistence region (below the surface). Notably, the eutectic point *E* corresponds to the system’s lowest co-melting temperature, far below the melting points of pure components (*t*_A_, *t*_B_, and *t*_C_) and critical for low-temperature phase change material design.

**Figure 3:**
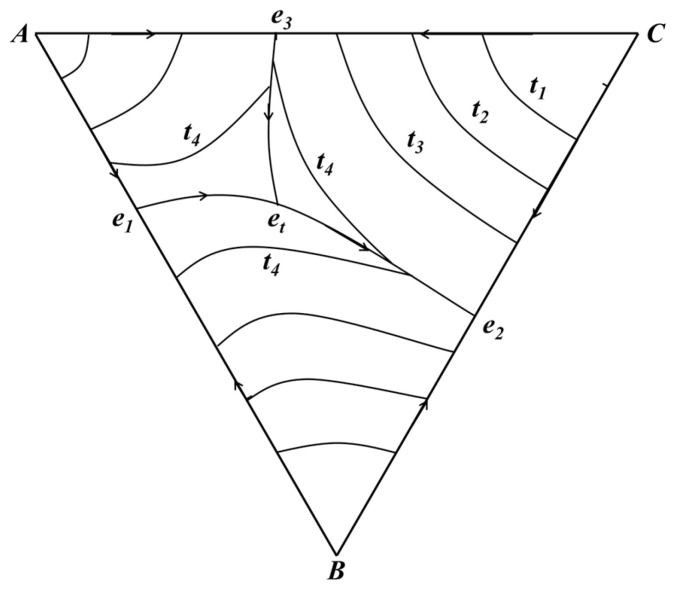
Projection of ternary system.

Figure 3 presents a 2D projection of the 3D ternary phase diagram in Figure 2, with the concentration triangle retained and temperature expressed by isotherms. The lines *e*_1_*e*_t_, *e*_2_*e*_t_, and *e*_3_*e*_t_ are projections of the binary eutectic lines, and *t*_1_, *t*_2_, *t*_3_, and *t*_4_ represent different isothermal lines. This projection intuitively illustrates the temperature variation trend of the system: temperatures decrease along the eutectic lines toward the ternary eutectic point *e*_t_, and isotherms partition the concentration triangle into distinct temperature zones, facilitating rapid evaluation of the phase state of any ternary composition at a given temperature.

For an ideal ternary system, plotting *T* against *x* using Equation (1) yields the melting curves. Based on these curves, and by calculating the eutectic channel curves of the ternary system, the phase equilibrium diagram of the ternary system under ideal conditions can be obtained. The eutectic channel curves refer to the connecting curves between individual binary eutectic points and the ternary eutectic point, such as curves *E*_1_*E*_t_, *E*_2_*E*_t_, and *E*_3_*E*_t_ in Figure 2. Along any of these channels, two solid phases are in equilibrium with the liquid phase.

Taking capric acid–lauric acid–myristic acid (CA-LA-MA) as an example, the phase diagram and thermal properties of fatty acid ternary eutectic mixtures are illustrated. For simplicity, let component 1 denote CA, component 2 denote LA, and component 3 denote MA. The melting points (*T*_m1_, *T*_m2_, *T*_m3_) and latent heats of fusion (Δ*H*_m1_, Δ*H*_m2_, Δ*H*_m3_) of CA, LA, and MA are listed in Table 1. The equations for the three eutectic channels are as follows:

(5) $$\left\{\begin{array}{l} x_{1}=\exp\left[\frac{\Delta H_{m1}}{R}\left(\frac{1}{T_{m1}}-\frac{1}{T_{m}}\right)\right]=\exp\left[\frac{176.0\times 172.26}{8.314}\left(\frac{1}{303.65}-\frac{1}{T_{m}}\right)\right]\\ x_{2}=\exp\left[\frac{\Delta H_{m2}}{R}\left(\frac{1}{T_{m2}}-\frac{1}{T_{m}}\right)\right]=\exp\left[\frac{182.0\times 200.32}{8.314}\left(\frac{1}{316.95}-\frac{1}{T_{m}}\right)\right]\\ x_{3}=\exp\left[\frac{\Delta H_{m3}}{R}\left(\frac{1}{T_{m3}}-\frac{1}{T_{m}}\right)\right]=\exp\left[\frac{188.6\times 228.37}{8.314}\left(\frac{1}{325.45}-\frac{1}{T_{m}}\right)\right] \end{array}\right.,$$

**Figure 4:**
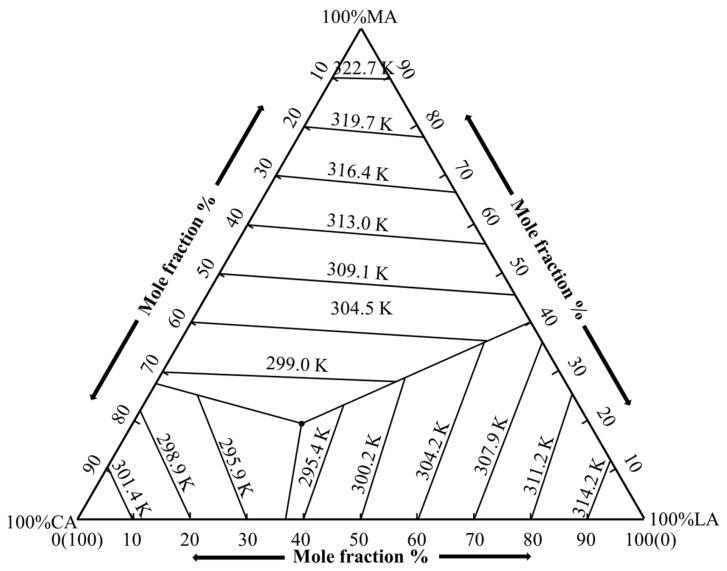
Phase diagram of the CA-LA-MA ternary eutectic mixtures.

In the above equations, *x*_1_, *x*_2_, and *x*_3_ represent the molar percentage compositions of CA, LA, and MA in the eutectic mixture, respectively, with *x*_1_ + *x*_2_ + *x*_3_ = 1. By plotting Equation (5) and connecting the corresponding temperatures on the binary phase diagrams and each pair of eutectic channels with straight lines to form individual isotherms, the theoretical ternary phase diagram of CA-LA-MA was obtained, as shown in Figure 4. In this diagram, the three vertices represent CA, LA, and MA, respectively. The isotherms (e.g., 299.0 K, 304.5 K) and eutectic channels are clearly displayed, with e being the eutectic point of the CA-LA-MA ternary system corresponding to a co-melting temperature of 290.26 K (the calculated result in Section 3.2). This diagram verifies the applicability of the ternary eutectic law to the target fatty acid system: the eutectic point e with low co-melting temperature is consistent with the structural characteristics of the ideal system in Figure 2, confirming the feasibility of developing low-temperature phase change materials based on the CA-LA-MA eutectic mixture.

### 3.2. Determination of Eutectic Point of Ternary Fatty Acid Mixtures by the Schroder Equation

The CA-LA-MA ternary system is still taken as an example to illustrate the determination method. The eutectic temperature Tm of ternary eutectic mixtures can be calculated using Equation (6), which is derived from Equation (1).

(6) $$1=\exp\left[\frac{\Delta H_{m1}}{R}\left(\frac{1}{T_{m1}}-\frac{1}{T_{m}}\right)\right]+\exp\left[\frac{\Delta H_{m2}}{R}\left(\frac{1}{T_{m2}}-\frac{1}{T_{m}}\right)\right]+\exp\left[\frac{\Delta H_{m3}}{R}\left(\frac{1}{T_{m3}}-\frac{1}{T_{m}}\right)\right],$$

To obtain the component xi at the eutectic point, simultaneous solution of Equations (1) and (4) is feasible. Specifically, substitute *T*_m1_, *T*_m2_, *T*_m3_, Δ*H*_m1_, Δ*H*_m2_, and Δ*H*_m3_ into Equation (6), respectively.

(7) $$\begin{array}{l} 1=\exp\left[\frac{176.0\times 172.26}{8.314}\left(\frac{1}{303.65}-\frac{1}{T_{m}}\right)\right]\\ +\exp\left[\frac{182.0\times 200.32}{8.314}\left(\frac{1}{316.95}-\frac{1}{T_{m}}\right)\right]+\exp\left[\frac{188.6\times 228.37}{8.314}\left(\frac{1}{325.45}-\frac{1}{T_{m}}\right)\right] \end{array},$$

The solution is obtained as follows: *T*_m_ = 290.26 K = 17.11 °C.

The eutectic composition xi (molar ratio) is derived from Tm and the Schroder equation (Equation (1)), as shown below:

(8) $$\left\{\begin{array}{l} x_{1}=\exp\left[\frac{\Delta H_{m1}}{R}\left(\frac{1}{T_{m1}}-\frac{1}{T_{m}}\right)\right]=\exp\left[\frac{170.6\times 172.26}{8.314}\left(\frac{1}{303.65}-\frac{1}{290.26}\right)\right]=0.5745\\ x_{2}=\exp\left[\frac{\Delta H_{m2}}{R}\left(\frac{1}{T_{m2}}-\frac{1}{T_{m}}\right)\right]=\exp\left[\frac{182.0\times 200.32}{8.314}\left(\frac{1}{316.95}-\frac{1}{290.26}\right)\right]=0.2803\\ x_{3}=\exp\left[\frac{\Delta H_{m3}}{R}\left(\frac{1}{T_{m3}}-\frac{1}{T_{m}}\right)\right]=\exp\left[\frac{188.6\times 228.37}{8.314}\left(\frac{1}{325.45}-\frac{1}{290.26}\right)\right]=0.1452 \end{array}\right.$$

Specifically, the theoretically calculated eutectic temperature of CA-LA-MA is 17.11 °C. At this temperature, the molar fraction ratio of the three individual fatty acids (CA:LA:MA) is 57.45:28.03:14.52, and the mass ratio is 52.58:29.81:17.61. The latent heat of fusion under this condition is calculated using Equation (4):

(9) $$\begin{array}{cc} \Delta H_{m} & =T_{m}\sum_{i=1}^{n}\left(\frac{x_{i}\Delta H_{mi}}{T_{mi}}\right)\\ & =290.26*\left(\begin{array}{l} \frac{0.5745\times 170.6\times 172.26}{303.65}+\frac{0.2803\times 182.0\times 200.32}{316.95}\\ +\frac{0.1452\times 188.6\times 228.37}{325.45} \end{array}\right),\\ & =31,569.6(J\cdot {\mathrm{mol}}^{-1})\;=167.8(J\cdot g^{-1}) \end{array}$$

Similarly, the eutectic mass ratio, melting point, and latent heat of fusion at the eutectic point of other ternary eutectic fatty acid mixtures can be calculated, with the results summarized in Table 2. As can be seen from the table, the calculated melting points of the ternary eutectic fatty acid mixtures range from 17.11 °C to 37.61 °C, and their latent heats of phase change are between 167.8 J·g^−1^ and 189.6 J·g^−1^.

### 3.3. Determination of Eutectic Point of Ternary Fatty Acid Mixtures by the “Pseudo-Binary” Method

**Figure 5:**
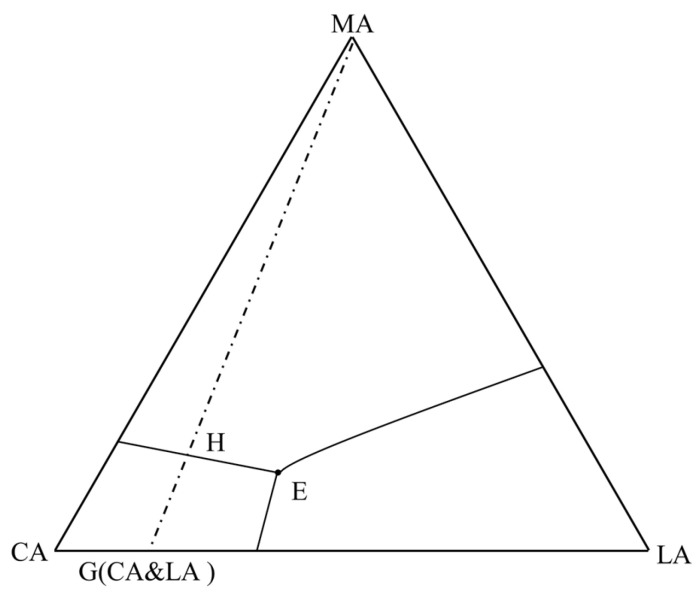
Phase diagram with top view of the CA-LA-MA.

**Figure 6:**
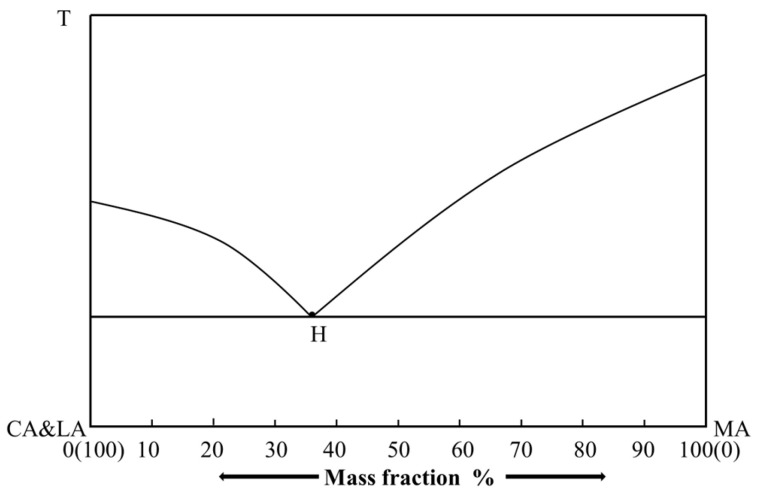
Phase diagram of pseudo binary system (CA&LA)-MA.

The eutectic point of the ternary mixture was determined using the “Pseudo-Binary” Method, and the CA-LA-MA ternary mixture is employed as an illustrative example herein. Specifically, a binary CA&LA mixture was first prepared following the proportions reported in Reference [7], with its concentration defined as x (point G in Figure 5). This mixture was treated as an integral component (CA&LA, a hypothetical pure substance), and the phase diagram of the CA&LA-MA “pseudo-binary” system was calculated following the standard procedure for binary phase diagrams, as illustrated in Figure 6.

**Figure 7:**
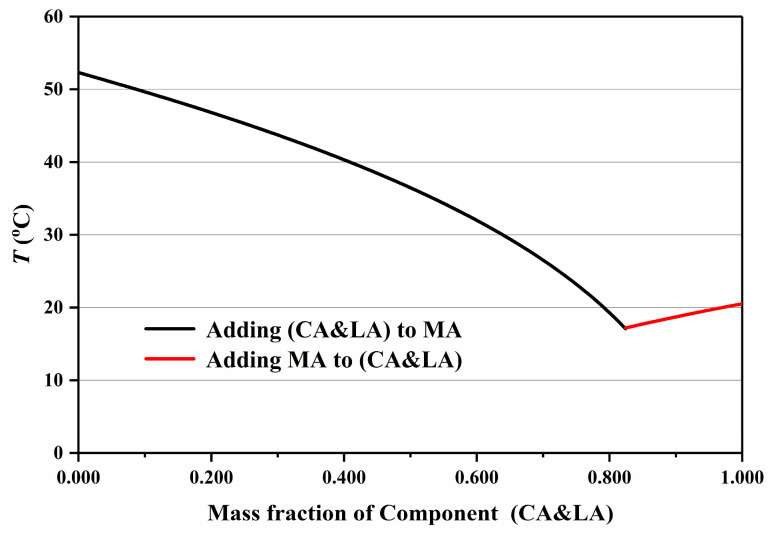
Phase diagram of (CA&LA)-MA pseudo-binary fatty acid eutectic mixture.

**Figure 8:**
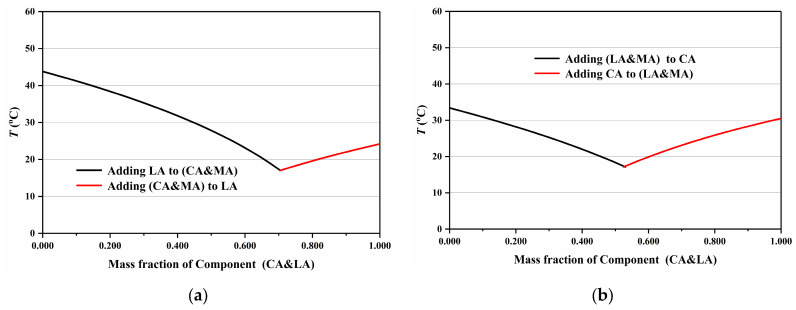
Phase diagram of (CA&MA)-LA (**a**) and CA-(LA&MA) (**b**) pseudo-binary fatty acid eutectic mixtures.

Based on Equations (3) and (4), the eutectic mass ratio, melting eutectic temperature, and phase change latent heat of the CA&LA eutectic mixture can be calculated. The same methodology can be extended to determine the relevant parameters of the CA&MA and LA&MA eutectic mixtures, with all results summarized in Table 3. By substituting the performance parameters of CA&LA and MA into Equations (1) and (2), the melting points and phase change latent heats of the ternary fatty acid system (CA&LA)-MA under different (CA&LA)-to-MA mass ratios can be calculated, as shown in Table 4, and the corresponding phase diagram is plotted in Figure 7. It can be observed from Table 4 and Figure 7 that at the eutectic point of the CA&LA-MA pseudo-binary system, the mass ratio of CA&LA to MA is 82.4:17.6, with the eutectic temperature being 17.20 °C and the phase change latent heat remaining 180.0 J/g (as shown in Table 5). Similarly, based on the performance parameters of CA&MA and LA&MA provided in Table 3, the eutectic points and corresponding performance parameters of the (CA&MA)-LA and CA-(LA&MA) pseudo-binary systems can be calculated, respectively (as shown in Table 5), with their phase diagrams plotted (as presented in Figure 8). As shown in Table 5, the eutectic point parameters of the three pseudo-binary fatty acid eutectic mixtures—(CA&LA)-MA, (CA&MA)-LA, and CA-(LA&MA)—are very close to those of the ternary eutectic mixture CA-LA-MA. This confirms that the pseudo-binary method is applicable for determining the eutectic point of ternary eutectic fatty acids.

### 3.4. Thermal Properties of Ternary Fatty Acid Mixtures

This section takes the CA-LA-MA ternary eutectic fatty acid as an example for illustration.

#### 3.4.1. DSC Analysis

**Figure 9:**
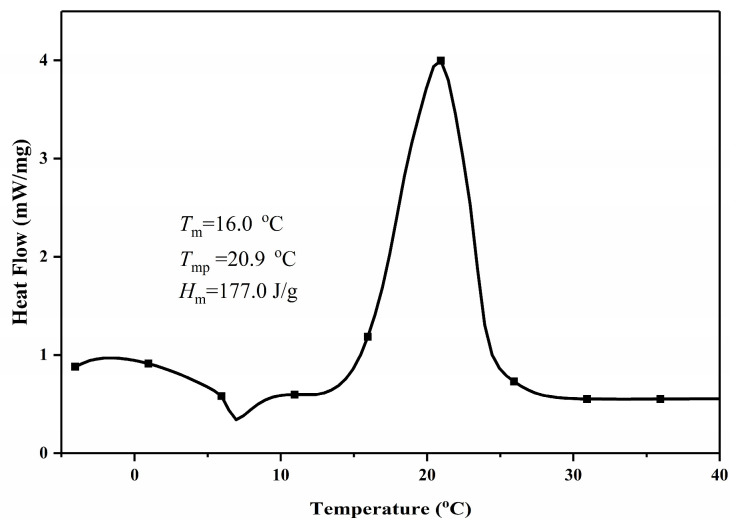
DSC curve of CA-LA-MA ternary eutectic mixture.

A series of mixtures with different mass ratios near the theoretically calculated eutectic point of the CA-LA-MA ternary eutectic mixture were prepared. The phase change temperatures of these mixtures were measured by DSC, and the mass ratio corresponding to the eutectic point was determined through comparison. The measured mass ratio of the eutectic point is CA:LA:MA = 52.0:30.0:18.0. The DSC curve of the CA-LA-MA ternary eutectic mixture at this mass ratio is shown in Figure 9. A comparison of the thermal performance parameters between the theoretical mass ratio and the experimental mass ratio is presented in Table 6. As shown in Figure 9, the CA-LA-MA ternary eutectic system exhibits a melting point of 16.0 °C and a phase change latent heat of 177.0 J·g^−1^, thus demonstrating excellent suitability for low-temperature scenarios including cold chain logistics. A comparative analysis between the experimentally determined thermal performance parameters and the theoretically calculated values is listed in Table 6. As shown in Table 6, minor discrepancies exist between the theoretical and experimental eutectic parameters of the CA-LA-MA ternary system: a 1.11 °C deviation in phase change temperature, a 9.2 J·g^−1^ difference in phase change latent heat (corresponding to a 5.48% relative error), and a slightly higher mass fraction of MA in the experimental eutectic composition. These discrepancies originate from two key aspects of theoretical modeling: (1) the ideal solution assumption inherent in the Schrödder equation, which fails to capture polar interactions and hydrogen bonding between CA, LA, and MA molecules, leading to deviations in predicted composition and temperature; (2) the exclusion of additional intermolecular interaction energy in calculations, resulting in an underestimation of latent heat.

To verify the reliability of the results in this study, we integrated literature comparison data into Table 6. It should be noted that public research on the CA-LA-MA system remains limited, with no other relevant reports retrieved except for Reference [32]. Our experimentally determined eutectic temperature (16.0 °C) shows a relative deviation of only 3.0% from the theoretical temperature of 16.5 °C reported in Reference [32], indicating good consistency in temperature measurements. In contrast, the latent heat value of 85.76 J·g^−1^ in Reference [32] exhibits a substantial relative deviation of 51.5% from our experimental result of 177.0 J·g^−1^; this value is significantly lower than the typical latent heat range (130–180 J·g^−1^) for CA-LA-MA ternary fatty acid eutectics. Even its alternative theoretical latent heat (131.4 J·g^−1^) deviates by 25.8% from our data, which is closer to the reasonable fluctuation interval. Our experimental data presented in the table are highly consistent with the basic regularity of similar systems, and the deviation from the relatively reasonable latent heat data in Reference [32] falls within an interpretable range, which corroborates the stability of our experimental results. Beyond theoretical modeling limitations, minor discrepancies between our experimental data and literature values are additionally attributed to fluctuations in raw material purity and differences in DSC testing conditions (e.g., heating/cooling rates). Overall, the magnitude of all consistent discrepancies is negligible for practical applications, and the strong agreement in temperature results across theoretical predictions, experiments, and available literature further confirms the validity of our experimental method and the stability of the CA-LA-MA eutectic system.

#### 3.4.2. FT-IR Spectroscopy Analysis

**Figure 10:**
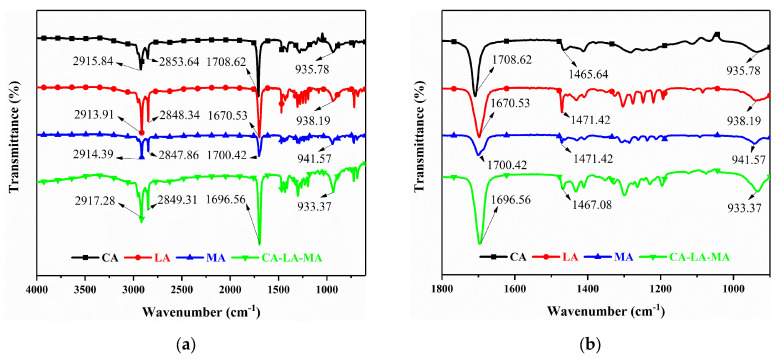
FT–IR curves of CA, LA, MA and CA-LA-MA. (**a**) Full-range FT-IR spectrum (400–4000 cm^−1^); (**b**) Magnified FT-IR spectrum (900–1800 cm^−1^).

Figure 10 shows the FT-IR spectra of CA, LA, MA and the CA-LA-MA ternary eutectic mixture. In the spectrum of the eutectic system, the peaks at 2917.28 cm^−1^ and 2849.31 cm^−1^ correspond to the asymmetric and symmetric stretching vibrations of -CH_2_- in the long alkyl chains of fatty acids. The peak at 1696.56 cm^−1^ is the stretching vibration of carboxyl C=O, and the slight shift of this peak indicates the formation of hydrogen bonds during the eutectic process. The peak at 1470.43 cm^−1^ corresponds to the bending vibration of -CH_2_-, which can reflect the packing state of alkyl chains. The peaks at 1298.71 cm^−1^ and 933.37 cm^−1^ are the stretching vibrations of carboxyl C-O. The peaks at 722.78 cm^−1^ and 687.62 cm^−1^ correspond to the rocking vibrations of long-chain -CH_2_- (n ≥ 4). These characteristic peaks are consistent with those of pure CA, LA and MA, indicating that the eutectic process retains the basic functional group structure of the raw materials, while intermolecular interactions are formed between components.

#### 3.4.3. Thermal Stability Analysis

**Figure 11:**
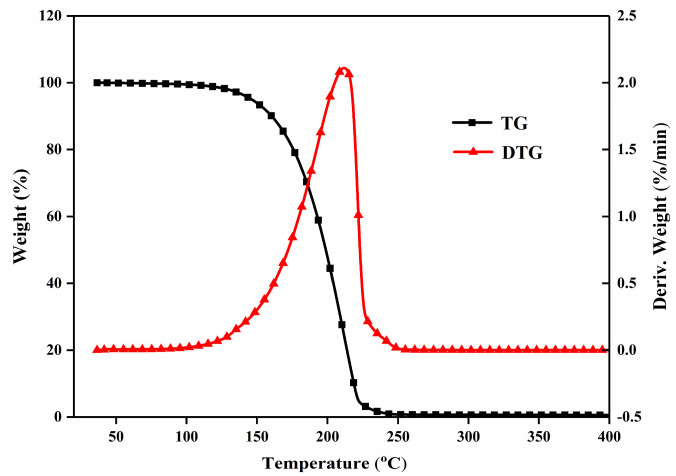
TGA curves of CA-LA-MA.

Figure 11 presents the TGA (black curve) and DTG (red curve) profiles of the CA-LA-MA ternary eutectic mixture. The initial weight loss (≈1% mass reduction) starts at 115.6 °C, which corresponds to the evaporation of trace adsorbed water in the sample (consistent with the hygroscopicity of fatty acid-based materials). The extrapolated onset temperature of major weight loss is 164.8 °C, marking the start of the sample’s thermal decomposition/volatilization process. The 130–210 °C interval is identified as the main weight loss region: within this range, the mass of the eutectic mixture decreases sharply (DTG curve peaks at 210.4 °C, corresponding to the maximum weight loss rate). By 210 °C, the total weight loss reaches nearly 98.5%, indicating that the eutectic components (CA, LA, MA) have almost completely volatilized (rather than decomposed)—this aligns with the low boiling point characteristics of short-chain fatty acids. Combined with the thermal behavior above, the CA-LA-MA ternary eutectic mixture is only suitable for low-temperature phase change energy storage systems with operating temperatures below 100 °C. This temperature limit ensures that the material remains stable (without weight loss or volatilization) during long-term service. For a more comprehensive evaluation, we compared our system’s thermal stability with similar eutectic systems in the literature: Ref. [35] investigated a high-temperature CaCl_2_-LiCl mixture (onset melting ~ 488 °C) but noted weak thermal stability (mass changes via decomposition) at elevated temperatures; Ref. [36] reported a LiCl-LiOH mixture (melting 269–292 °C) with good stability up to 500 °C. By contrast, our binary fatty acid eutectic (targeting ~ 16 °C) shows negligible mass change within its application temperature range, which aligns with its low-temperature thermal energy storage design.

## 4. Conclusions

In this work, capric acid (CA), lauric acid (LA), myristic acid (MA), palmitic acid (PA), and stearic acid (SA) were employed as alternative feedstocks to perform theoretical analysis on ternary eutectic systems composed of fatty acids. The phase diagrams corresponding to these ternary eutectic systems were established, and theoretical eutectic points were computed before being validated through experimental measurements. Subsequent experimental investigations focused on the thermal performance, molecular structure, and thermal stability of the prepared eutectic blends. Key findings of this study are summarized below:

(1) Theoretical eutectic points of the fatty acid-based CA-LA-MA ternary eutectic system were calculated, and the phase diagram of this ternary mixture was plotted. Minor deviations were observed between the theoretical and measured values, which may be attributed to trace impurities in the individual fatty acid reagents and experimental operational errors.

(2) A method for constructing phase diagrams of fatty acid ternary eutectic systems was developed, and the theoretical eutectic parameters of ten such eutectic blends were determined. The melting temperatures of these eutectic mixtures spanned 17.1 °C to 37.61 °C, with phase change latent heat values ranging from 167.8 J·g^−1^ to 189.6 J·g^−1^.

(3) The CA-LA-MA ternary eutectic mixture was synthesized, and its eutectic point was experimentally confirmed. Comprehensive characterization was conducted on its thermal properties, structural features, and thermal stability. For the CA-LA-MA system with a mass ratio of 52.0:30.0:18.0, the measured eutectic melting point was 16.0 °C, accompanied by a phase change latent heat of 177.0 J·g^−1^. Within its intended operating temperature range, this eutectic mixture demonstrates reliable thermal stability.

Given their moderate melting temperature (17.1–37.61 °C) and high latent heat (167.8–189.6 J·g^−1^), these fatty acid ternary eutectics show promising potential in low-temperature thermal storage scenarios, such as building energy conservation, electronic device thermal management, and solar heat storage.

## Figures and Tables

**Table 1:** Heat performances of CA, LA, MA, PA and SA.

PCM	*T*_m_ (°C)	*T*_mp_ (°C)	Δ*H*_m_ (J·g^−1^)	*T*_f_ (°C)	*T*_fp_ (°C)	Δ*H*_f_ (J·g^−1^)	*C*_p,s_ (J·(g·K)^−1^)	*C*_p,l_ (J·(g·K)^−1^)
CA	30.5	32.3	176.0	30.5	28.6	172.9	1.9	2.1
LA	43.8	46.5	182.0	41.9	40.0	190.6	1.7	2.3
MA	52.3	54.6	188.6	51.6	49.0	193.1	1.7	2.4
PA	54.3	56.9	206.5	52.6	50.3	194.4	1.9	2.8
SA	62.2	63.5	214.3	61.0	59.2	211.2	1.6	2.2

**Table 2:** Theoretical eutectic point of the CA-LA-MA ternary eutectic mixtures.

Ternary Eutectic Fatty Acid Mixtures	Theoretical Eutectic Mass Ratio	*T*_m_ (°C)	Δ*H*_m_ (J·g^−1^)
CA-LA-MA	52.58:29.81:17.61	17.11	167.8
CA-LA-PA	57.64:31.92:12.44	18.43	170.4
CA-LA-SA	59.28:34.36:6.36	19.61	170.3
CA-MA-PA	62.12:22.54:15.34	21.56	173.1
CA-MA-SA	66.85:24.87:8.28	23.00	173.1
CA-PA-SA	71.17:19.37:9.46	24.70	177.3
LA-MA-PA	46.44:30.56:23.00	29.06	177.9
LA-MA-SA	51.52:34.52:13.96	31.13	178.0
LA-PA-SA	54.72:29.52:15.76	33.06	184.1
MA-PA-SA	43.35:36.34:20.31	37.61	189.6

**Table 3:** Theoretical eutectic point of fatty acid binary eutectic mixtures.

Binary Eutectic Mixtures	Theoretical Eutectic Mass Ratio	*T*_m_ (°C)	Δ*H*_m_ (J·g^−1^)
CA&LA	63.1:36.9	20.52	184.0
CA&MA	72.2:27.8	24.19	177.0
LA&MA	59.4:40.6	33.39	187.0

**Table 4:** Phase change temperature and latent heat in pseudo-binary fatty acid eutectic mixtures.

Proportion of (CA&LA) (%)	Proportion of MA (%)	*T*_m_ (°C)
100.0	0.0	20.52
92.0	8.0	19.09
89.5	10.5	18.63
84.8	15.2	17.68
82.4	17.6	17.20
80.1	19.9	19.20
69.9	30.1	26.57
54.9	45.1	34.35
30.0	70.0	43.72
4.8	95.2	51.04
0.00	100	52.30

**Table 5:** Theoretical eutectic point of pseudo-binary fatty acid eutectic mixtures.

Fatty Acid	Mass Ratio	Mass Ratio	*T*_m_ (°C)	Δ*H*_m_ (J·g^−1^)
(CA&LA)-MA	(CA&LA):MA = 82.4:17.6	CA:LA:MA = 52.0:30.4:17.6	17.20	180.0
(CA&MA)-LA	(CA&MA):LA = 70.4:29.6	CA:LA:MA = 41.8:29.6:28.6	16.99	181.0
CA-(LA&MA)	CA:(LA&MA) = 52.5:47.5	CA:LA:MA = 52.5:28.2:19.3	17.12	182.0
CA-LA-MA	—	CA:LA:MA = 52.58:29.81:17.61	17.11	167.8

**Table 6:** Parameters of CA-LA-MA eutectic system: theoretical, experimental, and literature data.

Data Category	Composition (CA:LA:MA, wt %)	*T*_m_ (°C)	Δ*H*_m_ (J·g^−1^)
This Study—Theoretical	52.07:30.56:17.37	17.11	167.8
This Study—Experimental	52.0:30.0:18.0	16.0	177.0
Reference [32]—Theoretical	56.39:26.72:16.89	16.5	85.76
Reference [32]—Experimental	66.35:20.62:13.03	15.83	131.4

## Data Availability

The original contributions presented in this study are included in the article. Further inquiries can be directed to the corresponding authors.
